# The Sensitivity of the Method Used to Detect Atrial Fibrillation in Population Studies Affects Group-Specific Prevalence Estimates: Ethnic and Regional Distribution of Atrial Fibrillation in the REGARDS Study

**DOI:** 10.2188/jea.JE20081032

**Published:** 2009-07-05

**Authors:** Ronald J Prineas, Elsayed Z Soliman, George Howard, Virginia J Howard, Mary Cushman, Zhu-Ming Zhang, Claudia S Moy

**Affiliations:** 1 Department of Epidemiology and Prevention, Wake Forest University School of Medicine, Winston-Salem, NC, USA; 2 Departments of Biostatistics, School of Public Health, University of Alabama, Tuscaloosa, AL, USA; 3 Departments of Epidemiology, School of Public Health, University of Alabama, Tuscaloosa, AL, USA; 4 Department of Medicine, University of Vermont College of Medicine, Burlington, VT, USA; 5 National Institute of Neurological Disorders and Stroke, National Institutes of Health, Bethesda, MD, USA

**Keywords:** atrial fibrillation, race/ethnicity, REGARDS study

## Abstract

**Background:**

Among African-Americans, and in southern US states, the rates of stroke are high but the reported prevalences of atrial fibrillation (AF) are low. We hypothesized that the reported ethnic and regional distributions of AF are affected by the sensitivity of the methods that were used to detect AF in previous reports.

**Methods:**

A total of 18 833 black and white participants from the US national REasons For Geographic And Racial Differences In Stroke (REGARDS) study were included in this analysis. Levels of sensitivity to detect AF, from least to most sensitive, were created for combinations of self-report (SR) and ECG methods, as follows: (1) SR plus ECG, (2) ECG alone, (3) SR alone, and (4) SR or ECG. Geographic regions were dichotomized as Stroke Belt (the southern US states) and non-Stroke Belt. Logistic regression analysis estimated the odd ratios of AF associated with the Stroke Belt and black ethnicity for each diagnostic combination.

**Results:**

Residence in the Stroke Belt was significantly associated with AF when diagnosed by SR plus ECG (multivariable-adjusted OR, 0.66; 95% CI, 0.47 to 0.92), but not when diagnosed with SR or ECG (OR, 0.95; 95% CI, 0.85 to 1.06). Similarly, for the 4 methods used to detect AF, the strength of the association between black ethnicity and AF progressively decreased with increasing test sensitivity (ORs: 0.20, 0.40, 0.70, 0.71, respectively).

**Conclusions:**

The association of AF with residence in the Stroke Belt and black ethnicity was inversely related to the sensitivity of the method used to detect AF: as test sensitivity increased, the association became attenuated. This may partially explain the lower reported prevalence of AF in populations and regions with higher stroke rates.

## INTRODUCTION

Atrial fibrillation (AF) is the most common arrhythmia which currently affects 2.3 million Americans.^1^ It is associated with a more than 5-fold increase in stroke incidence, a relative risk that is the highest of the traditional risk factors for incident stroke.^2^ Because of this strong association between AF and the risk of stroke, it has been suggested that the ethnic and regional differences in stroke burden might be explained in part by the ethnic and regional differences in the prevalence of AF. However, despite the increased stroke burden among blacks^3^ and in the southern United States (the Stroke Belt),^4^^,^^5^ it has been reported that the prevalence of AF among blacks is lower than that among whites,^1^^,^^6^^–^^12^ and that there is no difference in AF prevalence between the Stroke Belt and any other region of the United States.^13^ Such a paradox warrants examination of the methods by which these studies reached their conclusions regarding ethnic and regional differences in AF prevalence. Previous studies have acknowledged the limitations of the 12-lead electrocardiogram (ECG) and self-report (SR) in detecting all AF cases—especially paroxysmal AF, which represents over 30% of cases^14^^–^^16^—but it was assumed that these limitations in the detection of AF would not affect the proportionate distribution of AF prevalence for different ethnicities and regions. This opinion was based on the assumption that the ability or inability (sensitivity) to detect AF would affect AF prevalence in all groups equally and that the resulting proportionate distribution of AF among groups would be unaffected. In the present analyses, we examine this assumption by estimating the prevalence of AF for the different levels of test sensitivity that exist among the diagnostic methods typically used to detect AF.

## METHODS

The REasons for Geographic And Racial Differences in Stroke (REGARDS) study is a US national, population-based, longitudinal study of black and white adults aged at least 45 years. The study was designed to oversample African Americans so that they represented 42% of the cohort, and to provide approximately equal numbers of men and women (45%/55%). By design, 50% of participants resided in the 8 southern US states referred to as Stroke Belt states (North Carolina, South Carolina, Georgia, Alabama, Mississippi, Tennessee, Arkansas, and Louisiana), and 50% resided in the other 40 contiguous states. Individuals were recruited from commercially available lists of residents by using a combination of mail and telephone contact. For those who agreed to participate, demographic information, medical history, and measures of cognitive function and quality of life were obtained by computer-assisted telephone interview. Standardized physical measures were collected at an in-home physical examination that included height and weight to calculate body mass index (BMI) in kg/m^2^, blood pressure, and resting ECG. The study methods were reviewed and approved by the Institutional Review Boards at the participating centers. Additional methodological details are provided elsewhere.^17^

Three to 4 weeks after the telephone interview, the in-home exam was conducted in-person by a health care professional from Examination Management Services, Inc. (EMSI). The ECGs were sent to a central ECG reading center at Wake Forest University, where they were read and coded by electrocardiographers blinded to clinical data.

Hypertension was defined as a systolic blood pressure of at least 140 mm Hg, a diastolic blood pressure of at least 90 mm Hg, or use of antihypertensive drugs. Diabetes was defined as fasting blood glucose >126 mg/dL, a non-fasting blood glucose >200 mg/dL, or use of insulin or oral diabetic medication. In addition to ECG detection of AF, a history of AF by SR was also recorded. AF by SR was defined as a positive response to the question: “*Has a physician or a health professional ever told you that you had atrial fibrillation?*” AF by ECG and/or SR was further classified into 4 groups: (1) AF by SR and ECG (SR plus ECG), (2) AF by ECG alone (ECG AF), (3) AF by SR alone (SR AF), and (4) AF by SR or ECG (SR or ECG). We developed this classification to provide different degrees of sensitivity to detect AF, so that AF by SR plus ECG would be the least sensitive and AF by SR or ECG would be the most sensitive.

Between January 25, 2003 and November 30, 2005, a total of 191 028 telephone numbers had been called for the purpose of recruiting participants, after which the database was closed for the purposes of this analysis. As defined by the standards recommended by Morton et al,^18^ the response rate (percentage of candidates that agreed to be interviewed among known eligible candidates contacted, plus an adjustment for estimate of likely eligible participants among unknown eligible participants) was 40.7% (31 556/77 526).^19^ As of December 1, 2005, there were 20 677 participants for whom in-home baseline data was complete. After exclusion of candidates with ECG evidence of an implanted pacemaker and those with poor quality ECG recordings, 18 833 participants remained for the present analysis.

### Statistical analysis

Frequency distributions of all variables were first inspected to identify anomalies and outliers possibly caused by measurement artifacts. Descriptive statistics included mean, SD, and percentiles for continuous variables, and frequency and percentage for categorical variables. Logistic regression models were used to estimate the associations, expressed as odds ratios, between AF (as measured using the different detection methods) and either ethnicity (black vs white) or geographic region (Stroke Belt vs non-Stroke Belt). Three models were fitted: model 1 unadjusted; model 2 adjusted for age, sex, and either ethnicity (for region analysis) or region (for ethnicity analysis); and model 3 adjusted for age, sex, hypertension, diabetes, BMI, and either ethnicity (for region analysis) or region (for ethnicity analysis).

## RESULTS

Table 1 shows the characteristics of the study population (*n* = 18 833), as well as the unadjusted prevalence of AF, stratified by ethnicity and geographic region. The average age of the study population was 65.9 years; 41% were blacks, 51.5% were women, 57.5% had hypertension, and 21.2% were diabetics. Unadjusted AF prevalence in all subgroups varied markedly across the levels of sensitivity created by the different combinations of SR- and ECG-diagnosed AF. AF detected by SR or ECG was the most sensitive, followed by SR alone, ECG alone, and SR plus ECG. In general, AF detected by SR or ECG was more than 7 times as prevalent as AF by SR plus ECG: AF prevalence in the total population by SR or ECG was 7.8%, as compared to only 0.8% when AF was detected by SR plus ECG. Regardless of these variations in sensitivity, the unadjusted prevalence of AF in the Stroke Belt did not differ from that of any other region of the United States. AF was more prevalent in whites than in blacks.

**Table 1.** Characteristics of the study population and prevalence (%) of atrial fibrillation (AF), stratified by ethnicity and geographic region

Variable	Entire population *n* = 18 833	Region		Ethnicity	
	
		Stroke Belt *n* = 9786	Non-Stroke Belt *n* = 032	Blacks *n* = 7722	Whites *n* = 11 105
Age (years)	65.9 ± 9.0	65.4 ± 8.9	66.5 ± 9.0	65.3 ± 8.9	66.3 ± 9.0
Females (%)	51.5	52.1	50.8	59.6	45.8
Blacks (%)	41.0	34.8	47.8	100.0	0.0
Body Mass Index (wt/ht^2^)	29.2 ± 6.1	29.1 ± 6.1	29.3 ± 6.1	30.6 ± 6.7	28.2 ± 5.5
Hypertension (%)	57.5	57.8	57.2	69.4	49.2
Diabetes (%)	21.2	21.8	20.7	29.6	15.5
AF (%)					
​ SR plus ECG	0.8	0.7	0.9	0.2	1.2
​ ECG	1.4	1.3	1.5	0.7	1.8
​ SR	7.2	7.3	7.1	6.3	7.8
​ SR or ECG	7.8	7.8	7.8	6.8	8.5

*SR = Self-reported; ECG = Electrocardiogram.

Table 2 shows the unadjusted and multivariable-adjusted associations of ethnicity and region with AF, by the 4 levels of sensitivity. In all models, the associations—expressed as odds ratios—of region (Stroke Belt vs non-Stroke Belt) and ethnicity (blacks vs whites) with AF showed an inverse relation with the sensitivity to detect AF, ie, the higher the sensitivity of the detection method, the lesser the effect of ethnicity or region. The effect of region on the prevalence of AF was statistically significant when AF was diagnosed using the less sensitive methods, SR plus ECG and ECG alone (ORs [95% CI] for model 3, 0.66 [0.47 to 0.92] and 0.71 [0.55 to 0.92], respectively), but was nonsignificant when AF was diagnosed by the more sensitive methods, SR and SR or ECG (ORs for model 3, 0.96 [0.85 to 1.08] and 0.95 [0.85 to 1.06], respectively). Similarly, the association of ethnicity with AF was progressively attenuated when AF was measured by progressively more sensitive detection methods (ORs for all models, 0.20 [0.12 to 0.33], 0.40 [0.29 to 0.54], 0.70 [0.62 to 0.79], 0.71 [0.63 to 0.80]).

**Table 2.** Unadjusted and multivariable-adjusted logistic regression analysis of the association of atrial fibrillation (AF) with ethnicity and geographic region, by sensitivity to detect AF*

Method to Detect AF		Belt vs non-Belt OR (95%CI)	Black vs white OR (95% CI)
SR plus ECG	Model 1	0.80 (0.58–1.10)	0.19 (0.12–0.32)
	Model 2	0.69 (0.50–0.95)	0.20 (0.12–0.33)
	Model 3	0.66 (0.47–0.92)	0.20 (0.12–0.33)
ECG	Model 1	0.83 (0.65–1.06)	0.39 (0.29–0.52)
	Model 2	0.77 (0.60–0.98)	0.43 (0.31–0.57)
	Model 3	0.71 (0.55–0.92)	0.40 (0.29–0.54)
SR	Model 1	1.02 (0.91–1.14)	0.80 (0.71–0.90)
	Model 2	0.99 (0.88–1.10)	0.78 (0.70–0.88)
	Model 3	0.96 (0.85–1.08)	0.70 (0.62–0.79)
SR or ECG	Model 1	1.01 (0.91–1.12)	0.80 (0.71–0.89)
	Model 2	0.98 (0.88–1.09)	0.79 (0.70–0.88)
	Model 3	0.95 (0.85–1.06)	0.71 (0.63–0.80)

*Model 1 = unadjusted; model 2 = adjusted for age, sex, and ethnicity (in region analysis) or region (in ethnicity analysis); model 3 = adjusted for model 2 variables, plus hypertension, diabetes, and BMI.

## DISCUSSION

The main purpose of this study was to explain the apparent contradiction between the reported increase in stroke burden in blacks, and in the Stroke Belt, versus the lower reported prevalence of AF in blacks, as compared to whites, and the absence of a difference in the prevalence of AF between the Stroke Belt and other regions in the United States. Our hypothesis was that the reported association of ethnicity and region with AF was much affected by the sensitivity of the current methods used for detecting AF. The present assumption is that a detection method with a low sensitivity would equally under-diagnose AF in all groups; thus, the proportionate distribution of AF between groups would be unaffected, and the effects of the groups on determinations of the risks of AF would be the same, regardless of the methods used to detect AF. However, the difficulty of estimating the prevalence of AF in large cohorts or population samples is mainly due to the difficulty of detecting paroxysmal AF (PAF), which is characterized by intermittently normal heart rhythm and ECG patterns between episodes. Hence, the ability to estimate the prevalence of AF in any population is related to the proportion of individuals with PAF. If groups differ in the prevalence or self-recognition of paroxysmal AF, the ability to detect AF will differ between groups. There may well be such a difference in the awareness of PAF symptoms—and of subsequent follow-up medical consultation—between different subgroups of the population. However, at present, no data on ethnic or regional differences in the prevalence of PAF have been published. Long-duration ECG recordings (24–48 hour Holter monitoring) would be a better way to accurately estimate the prevalence of PAF, but the high cost of such recordings is a major obstacle for large epidemiological studies.

The current study showed that there is an inverse relation between the sensitivity to detect AF and the association of ethnicity and region with AF, i.e., the more sensitive the method, the more attenuated the association of ethnicity and region with AF. As the method for detecting AF became more sensitive, the association of geographic region with AF became statistically nonsignificant and the effect of ethnicity was attenuated. These findings suggest that if a more sensitive approach for AF detection (such as long-term Holter monitoring) were employed, it might result in an estimate showing a reversed distribution of AF, with blacks having a higher, or at least equal, prevalence of AF, compared to whites. Unfortunately, long-term Holter monitoring data are not available and, as a result, the true prevalence of AF remains unknown. Such a hypothetical conclusion, despite being at odds with the results of less sensitive detection methods (SR and ECG) used by previous studies, accords with the high stroke burden in blacks and the strong association between AF and stroke.

It is noteworthy that, despite the progressive attenuation of the association between ethnicity and AF as sensitivity increased, the association remained statistically significant, which was not the case for the association between region and AF. These findings might be explained by the fact that the different levels of diagnostic sensitivity created in this analysis were based upon 2 methods that are known to have a low sensitivity to detect AF, at least in comparison to 24-Hour Holter monitoring and hospital-diagnosed AF. The observed attenuation of the effect of differences in ethnicity as an effect of using more sensitive methods for AF detection might be partially explained by the possibility that blacks may have more paroxysmal AF than whites. Thus, by using a more sensitive method, more paroxysmal AF would be detected and the difference in AF prevalence between blacks and whites would diminish. There is indirect evidence to support the notion that blacks have a higher prevalence of paroxysmal AF than whites. In a recent prospective study,^20^ metabolic syndrome was a significant risk factor for paroxysmal AF, and was independent of left atrial diameter and age (OR, 2.8; 95% CI, 1.3 to 6.2; *P* < 0.01). Also, because blacks have a high prevalence of the metabolic syndrome,^21^ they might be more likely to develop paroxysmal AF. Further, in a more recent report in which the ethnic distribution of electrocardiographic predictors of AF was used to make inferences about future ethnic distribution of AF, the propensity to develop AF in blacks was almost 3 times that of whites. The authors defended their findings by mentioning that the use of electrocardiographic predictors to make inferences about the ethnic distribution of AF would result in higher sensitivity and would not be affected by paroxysmal AF, the presence of which might have been masked by the methods traditionally used to detect AF in studies of the ethnic distribution of AF.^22^

### Limitations

Selection bias might have occurred due to exclusion of participants with a history of stroke, and to the possibility that participants who agreed to participate in this study may have been more or less sick than those who refused to participate. However, given the satisfactory participation rate (compared to studies with a similar design) and the large sample size, it is unlikely that such a limitation would have a major effect on our conclusions.

We did not test differences in participants’ understanding of the term “atrial fibrillation,” or whether inequities in access to proper healthcare might have resulted in less self-reported AF among blacks compared to whites. Nevertheless, detection of AF in the present analysis also included ECG, an objective method of AF detection.

### Conclusion

In summary, the current study showed that there is an inverse relation between the sensitivity of the method used to detect AF and the association of ethnicity and region with AF: more sensitive methods for detecting AF resulted in greater attenuation of the effect of ethnic and regional differences on prevalence estimates of AF. Given the known limitations in the sensitivity of the current methods used to detect AF, our results may partially explain the apparent contradiction of the low prevalence of AF in populations and regions known to have high rates of stroke.
